# Short and Long Term Behavioral and Pathological Changes in a Novel Rodent Model of Repetitive Mild Traumatic Brain Injury

**DOI:** 10.1371/journal.pone.0160220

**Published:** 2016-08-09

**Authors:** Kelly M. McAteer, Frances Corrigan, Emma Thornton, Renee Jade Turner, Robert Vink

**Affiliations:** 1 Adelaide Centre for Neuroscience Research, School of Medicine, The University of Adelaide, Adelaide, South Australia, Australia; 2 Sansom Institute for Health Research, The University of South Australia, Adelaide, South Australia, Australia; Uniformed Services University, UNITED STATES

## Abstract

A history of concussion, particularly repeated injury, has been linked to an increased risk for the development of neurodegenerative diseases, particularly chronic traumatic encephalopathy (CTE). CTE is characterized by abnormal accumulation of hyperphosphorylated tau and deficits in learning and memory. As yet the mechanisms associated with the development of CTE are unknown. Accordingly, the aim of the current study was to develop and characterize a novel model of repetitive mTBI that accurately reproduces the key short and long-term functional and histopathological features seen clinically. Forty male Sprague-Dawley rats were randomly assigned to receive 0, 1 or 3x mTBI spaced five days apart using a modified version of the Marmarou impact-acceleration diffuse-TBI model to deliver 110G of linear force. Functional outcomes were assessed six and twelve weeks post-injury, with histopathology assessed twenty-four hours and twelve weeks post-injury. Repetitive mTBI resulted in mild spatial and recognition memory deficits as reflected by increased escape latency on the Barnes maze and decreased time spent in the novel arm of the Y maze. There was a trend towards increased anxiety-like behavior, with decreased time spent in the inner portion of the open field. At 24 hours and 12 weeks post injury, repetitive mTBI animals showed increased tau phosphorylation and microglial activation within the cortex. Increases in APP immunoreactivity were observed in repetitive mTBI animals at 12 weeks indicating long-term changes in axonal integrity. This novel model of repetitive mTBI with its persistent cognitive deficits, neuroinflammation, axonal injury and tau hyperphosphorylation, thus represents a clinically relevant experimental approach to further explore the underlying pathogenesis of CTE.

## Introduction

In recent years, awareness of mild traumatic brain injury (mTBI), or concussion, has increased significantly at both local and international levels. It is particularly common in contact sports such as American Football (NFL), Australian Rules Football (AFL) and boxing. In the United States alone, an estimated 1.6–3.8 million sport or recreation related concussions occur each year, although this number may be much higher given that many individuals with a potential concussion do not seek further treatment [1]. The incidence of concussion in professional contact sports has also risen in recent years, with 73% of retired professional AFL players reporting that they experienced at least one concussion over their playing career, with over half reporting multiple concussions [2].

Concussion is defined as a complex pathophysiological process affecting the brain induced by traumatic biomechanical forces, caused by either a direct blow to the head, face or neck or via excessive force elsewhere on the body transmitted to the head [3]. The injury typically results in the rapid onset of acute neurological dysfunction and clinical symptoms such as loss of consciousness, dizziness, headache, and photophobia [3, 4]. Although concussion may result in neuropathological changes, there are typically no abnormalities observed within the concussed brain using standard structural imaging techniques and any clinical symptoms observed are the result of functional disturbances [3].

TBI, regardless of severity level, involves a complex series of biochemical changes within the brain involving neurotoxicity, metabolic imbalances and general disruption of ionic and cellular homeostasis [5]. Most concerning however is the state of vulnerability that these cells enter following a concussive event, where if another concussion is sustained during the vulnerable period, the damage to these cells exponentially increases and may become irreversible [6, 7]. However, increasing evidence of a link between repetitive mTBI and emergence of neurodegenerative disorders later in life, such as Alzheimer’s disease (AD) and more recently, chronic traumatic encephalopathy (CTE) emphasizes the potential danger of multiple concussions.

CTE is thought to be a progressive tauopathy, characterized by the deposition of tau protein in the form of neurofibrillary tangles (NFTs) within the superficial cortical layers of the frontal and temporal lobes [8]. It is believed that patients that are believed to be diagnosed with CTE display signs of short term memory dysfunction, difficulties in higher-order decision making such as planning and multitasking, apathy, emotional instability and depression [8, 9]. These clinical representations of CTE are postulated to develop from a progressive loss of neurons and inclusions of NFTs in the associated functional areas, including the frontal cortex, temporal lobe and hippocampus [9]. Although there are clinical and pathological signs suggestive of the presence of CTE within some individuals, the link between repeated mTBI and the later emergence of this pathology is yet to be fully elucidated [10].

In order to understand the pathophysiology of CTE, appropriate models that accurately represent key clinical and pathological features of repeated mTBI are required. In order model the type of brain injury that is currently observed in sports injuries, it has been proposed that a number of criteria should be met. These include that the head must be struck directly; that the impacts should occur with high velocity and rapid acceleration of the head, both rotational and angular [11–13]; that the injuries should be mild enough not to cause more severe injury such as edema, neuronal damage or hemorrhage [11, 14]; and that long-term manifestations of the injury should reflect the onset of CTE, including mild cognitive deficits, changes in mood and the appearance of neuropathological features such as tau phosphorylation [15–17]. The aims of the current study were to assess the effectiveness of the well characterized Marmarou impact acceleration model [18] in producing an *in vivo* model of repetitive mTBI that will accurately portray the effects seen clinically.

## Materials & Methods

### Animals

All experimental procedures were carried out in accordance with the recommendations in the Australian Code for the Care and Use of Animals for Scientific Purposes 8^th^ edition (2013), and approved by the University of Adelaide Animal Ethics Committee (M-2012-225). Forty male Sprague-Dawley rats were randomized into one of three experimental groups: sham surgery (no injury), single mTBI (1x injury) or repetitive mTBI (3x injuries). Injuries occurred five days apart over a ten-day period (Table 1), as based on previous work, with this spacing allowing for resolution of the inflammatory response between injuries, as would be seen at two-four weeks following a human concussion [19]. The study was further divided into two arms: 1) a 12-week behavioral outcome (n = 25) study, encompassing motor and cognitive components; and 2) examination of histology at 24 hours (n = 15) following final injury or sham surgery.

**Table 1:** Injury schedule for single and repetitive mild traumatic brain injury (TBI) animals.

Number of TBI	Day 0	Day 5	Day 10
**0 (SHAM)**	NO INJURY	NO INJURY	NO INJURY
**1**	NO INJURY	NO INJURY	**INJURY**
**3**	**INJURY**	**INJURY**	**INJURY**

### Induction of Mild Traumatic Brain Injury

Animals were injured using a modified version of Marmarou impact acceleration model of diffuse traumatic brain injury [18] titrated to produce a 110g average linear acceleration force [20]. Animals were anaesthetized with 5% isoflurane and the skull was exposed via a midline incision to adhere a stainless steel disc (10mm diameter, 3mm depth) centrally between the lambda and bregma sutures using a polyacrylamide adhesive. Animals were then secured to a foam bed (Type E bed foam, Foam to Size) and injury induced via the release of a 450g brass weight from a height of 1 meter directly onto the steel disc. Previous studies have shown that this injury produces an average linear acceleration force of 110g [20] which is that typically observed in human concussive impacts [21]. Following injury, the incision was closed using sutures, a local anesthetic applied (lignocaine) and the animal allowed to regain consciousness. Animals not receiving an injury on a scheduled injury day as per Table 1 received all surgical procedures without undergoing injury. All animals were weighed daily following commencement of the injury schedule.

### Functional Outcome Measures

Animals were assessed weekly for motor outcome using the rotarod test [22], while changes in locomotion, anxiety and spatial memory were assessed at six and twelve weeks post-injury using the Open Field, Y Maze and Barnes Maze functional outcome tests [23–25].

#### Rotarod

The accelerating rotarod is widely considered the most sensitive test of motor function following diffuse TBI [22]. The device consists of a rotating assembly of 18 metal rods, each with a diameter of 1 mm so as to introduce a grip component to the test, where the rotational speed of the assembly is gradually increased from 0 to 30 revolutions per minute over a 2-min period. To establish a baseline measure, animals were pre-trained on the rotarod to achieve the maximum running time of 120s for a four-day period pre-injury. The duration in seconds, up to a maximum of 120 secs, was recorded at the point when animals had either completed the task, clung to the rods for 2 consecutive rotations without actively walking or had fallen off. At 24 hours following each injury, then every seven days following the final injury, rats were assessed using the same criteria to determine any loss in motor function.

#### Open Field

The Open Field test is a common measure of anxiety in rodents [23]. It consists of a 1m x 1m box divided into 10cm x 10cm grids in which the animal is placed in the center and allowed to explore freely for five minutes, with the number of squares traversed, as well as the percentage of inner squares versus outer squares (the peripheral two rows) calculated. This test is based on the innate curiosity of the rat, where normal, uninjured animals normally will explore the entire area quite readily, whilst injured animals are anxious and will spend less time exploring the inner portion of the field.

#### Y Maze

The Y Maze assesses spatial and recognition memory in rodents, again based on the rodent’s innate curiosity and desire to explore new areas [24]. The three arms are arbitrarily assigned into start, novel and other arms. The animal is first introduced into the maze with the novel arm blocked off and allowed to freely explore for three minutes. One hour after the initial exposure the rat is reintroduced into the maze with all three arms open and allowed to explore freely for three minutes. This test works on the basis that an uninjured animal will spend more time exploring the novel arm to which they have not been previously exposed, rather than the other two arms. In order to remove scent trails the maze was wiped thoroughly with 70% ethanol after each trial. The experimenter was not in the room during the trials, with all trials captured on video. A preference index was calculated as time spent in the novel arm versus time spent in all three arms and analyzed.

#### Barnes Maze

The Barnes Maze assesses spatial learning and memory in rodents, taking advantage of the rodent’s innate behavior to escape from brightly lit open areas [25]. The apparatus consists of a circular maze 1.2m in diameter, with eighteen escape holes (5cm diameter) placed around the circumference, with an escape box located underneath one of these holes. An aversive stimulus in the form of a floodlight is introduced to motivate the animal into finding the escape box. The animal is placed in the center of the maze and if the animal discovers and enters the box within the 3 min test timeframe, the light is switched off and the animal is returned to its home cage. If the animal fails to find the escape box, or if the animal finds the escape box but does not enter within the allotted three minutes, it is gently guided to the box by the experimenter and held there for 20s with the light switched off before being returned to its home cage. Animals undertook 2 trials, 15 mins apart over a 4 day period. Escape latency was recorded as the time taken for the animal’s head to enter the escape box, with the average recorded across the 2 trials per day. Animals failing to successfully locate the escape box on any given day received the maximum score possible for that trial (180s).

### Immunohistochemistry

Following the completion of the 12 week behavioral outcome studies a random subset of animals (n = 6 per group), and for the histology subset at 24 hours (n = 5 per group), animals were sacrificed via transcardial injection of 10% buffered formalin. Brains were then removed and stored in 10% buffered formalin for 24 hours. At this stage all brains were examined for evidence of hemorrhage, contusions or overt edema. They were then sectioned into 11 x 2mm areas using a rodent brain matrix (Kopf) and embedded in paraffin wax. For each animal a section was cut from the region −4.5 mm from the bregma, as this was located directly underneath the impact site. Immunohistochemical staining for neuroinflammation (IBA_1_, rabbit polyclonal, 1:20,000, Wako Pure Chemical Industries, 019–19741), axonal injury (amyloid precursor protein (APP), mouse monoclonal, 1:1000, Novocastra, NCL-APP) and phosphorylated tau (AT180, mouse monoclonal, 1:1000, Thermo Fisher, MN1040) was undertaken. Prepared sections were dewaxed and endogenous peroxidases blocked with 0.01% hydrogen peroxide/methanol. Slides were then placed into a citrate retrieval solution and microwaved at 100°C for 10 minutes prior to blocking non-specific binding using normal horse serum and application of the appropriate primary antibody for overnight incubation at room temperature. Secondary antibody (Vector Laboratories, anti-mouse/anti-rabbit, 1:250) was then applied for 30 minutes, followed by streptavidin peroxidase conjugate (Sigma-Aldrich, 1:1000) for 1 hour. Bound antibody was detected via application of 3,3’-Diaminobenzidine (DAB, Sigma-Aldrich, D-5637) followed by counterstaining with hematoxylin.

All slides were scanned using digital Nanozoomer technology (Hamamatsu) and viewed using NDP view software (Hamamatsu). Sections stained with AT180 and IBA_1_ were examined in 1mm^2^ cortical areas directly beneath the impact site in each sample. Phosphorylated tau, identified as dark brown staining of the cytoplasm, was analyzed by counting the number of AT180 positive cells within the selected region of interest. IBA_1_ staining was assessed by counting the number of resting microglia, with lighter staining cell bodies and extended processes, as well as activated microglia, which had dark staining cell bodies and retracted, dark processes, and then calculating the ratio of resting versus activated microglia within the selected region in interest. APP staining was assessed using a rating scale for level of intensity in cortical, hippocampal (CA2) and thalamic regions. All histological assessments were undertaken by two independent observers blinded to the treatment groups.

### Statistical Analysis

All data were analyzed using GraphPad Prism^**®**^ statistical software. All immunohistochemical analyses excluding APP scoring, Barnes Maze and Rotarod were evaluated using a one-way analysis of variance (ANOVA) followed by Tukey post-hoc analysis. APP scoring data were evaluated using a Kruskal-Wallis test with Dunn’s post-hoc analysis. Barnes Maze and Rotarod data were evaluated using a repeated measures two-way ANOVA followed by Tukey post-hoc analysis. A p value of p<0.05 was considered significant. All results are expressed as mean ± SEM.

## Results

There were no adverse acute effects following induction of injury, with animals recovering well with an average of 0–10g of weight loss observed at 24 hours post-injury that was regained within 2–5 days.

### Functional Outcome

#### Cognition

**Fig 1:**
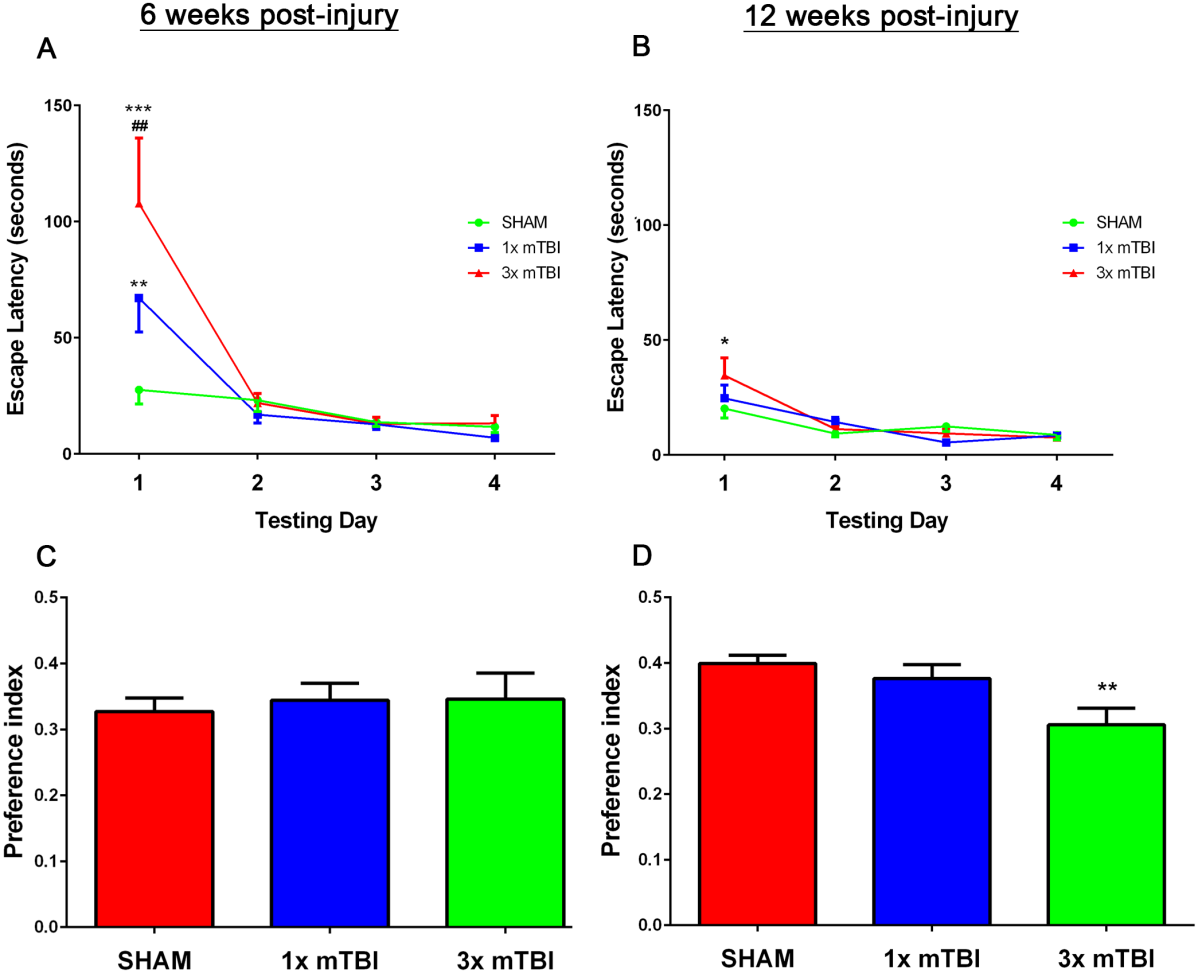
Behavioral Assessment post-injury. Barnes Maze acquisition phase trials at 6 weeks (A) and 12 weeks (B) post-injury. Y Maze testing at 6 weeks (C) and 12 weeks (D) post-injury (*p<0.05, **p<0.01 compared to sham animals, ##p<0.01, #p<0.05 compared to 1x mTBI animals, Sham, 1 x mTBI n = 9; 3x mTBI n = 7).

At 6 weeks post-injury, Barnes Maze testing on Day 1 showed increased latency in the acquisition phase in 3x mTBI (107.85±28.15, p<0.001) and 1x mTBI (67.17±14.73, p<0.01) animals when compared to shams (27.61±6.05) (Fig 1A). Similar results in 3mTBI animals were observed at 12 weeks post-injury in the acquisition phase, with a significant difference observed on day 1 testing between 3x mTBI (34.64±7.58) and sham (22±4.03) animals (p<0.05), whilst no increase in latency was observed in 1x mTBI (24.67±5.81) animals (Fig 1B). All injury groups returned to sham levels by day 2 of testing and remained at sham levels at both the 6 and 12 week time-points post-injury.

In the Y maze, there were no significant differences between groups at 6 weeks post-injury (Fig 1C). However at 12 weeks post-injury 3x mTBI animals demonstrated a significant decrease in spatial memory as shown by a decrease in preference index (p<0.05), compared to sham and 1x mTBI groups (Fig 1D).

#### Open Field

**Fig 2:**
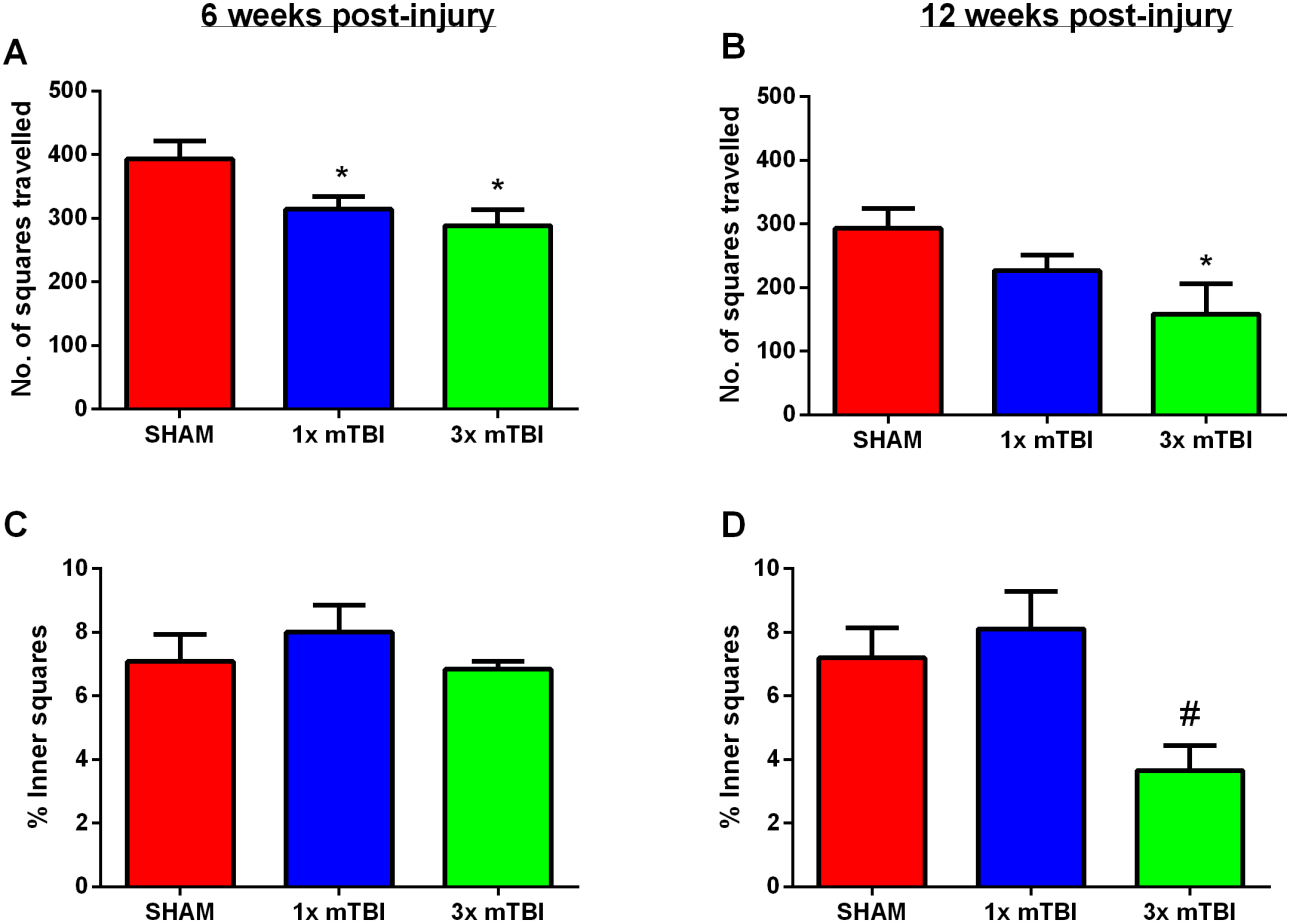
Open Field. Number of total line crossings 6 weeks (A) and 12 weeks (B) post-injury, and % of inner square line crossings compared to the total number at 6 weeks (C) and 12 weeks (D) post-injury. (*p<0.05 compared to sham, #p<0.05 compared to 1x mTBI animals; Sham, 1 x mTBI n = 9; 3x mTBI sham, 1 x mTBI n = 9; 3x mTBI n = 7)

In the Open Field, 3x mTBI animals demonstrated a significant decrease (p<0.05) in total line crossings at 6 weeks post-injury (288.43±25.4), as did the 1x mTBI animals (314.33±19.91) when compared to sham animals (393.56±27.8) (Fig 2A). Comparable results were also observed at 12 weeks post-injury in the 3x mTBI group, with a significant decrease (p<0.05) in open field exploration time (158.5±43.6) compared with sham animals (293.67±30.12) (Fig 2B). However, there were no differences observed between 1x mTBI animals and sham or 3x mTBI animals at this time point.

There were no differences in the percentage of inner squares versus total exploration of the open field at 6 weeks injury (Fig 2C). However at 12 weeks post-injury a significant group effect was found via one-way ANOVA (p<0.05), with post-hoc analysis reporting a trend between the sham and 3x mTBI groups (p = 0.09), with sham animals spending 7.2±2.5% of their time in the inner part of the field compared to 4.5±2.1% in 3x mTBI animals. 1x mTBI animals were similar to shams, with 8.1±3.1% of time spend in the inner part of the field, such that they were significantly different to 3x mTBI animals (p<0.05) (Fig 2D).

#### Motor Outcomes

**Fig 3:**
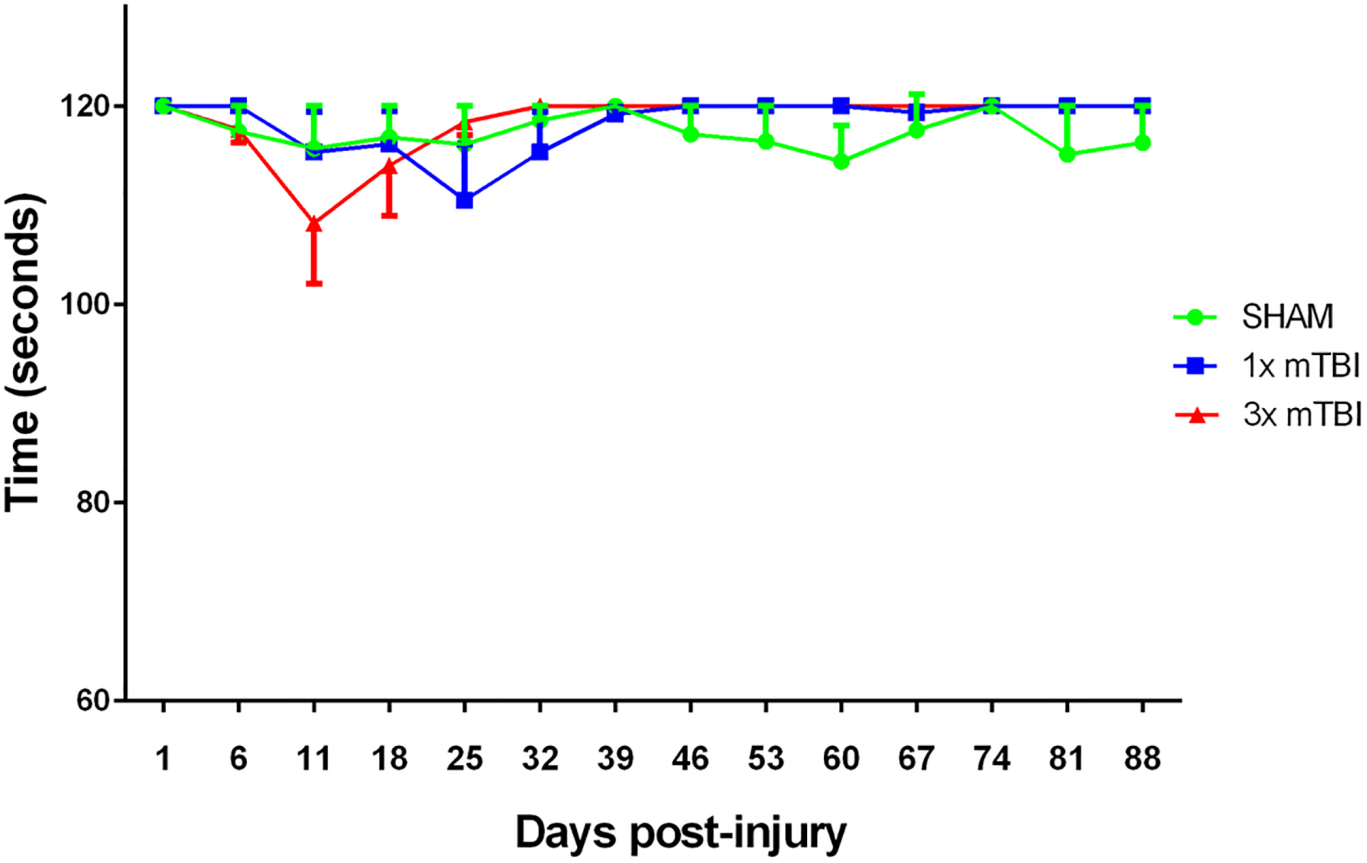
Motor Assessment post-injury. Weekly rotarod assessments showing no significant changes in motor function over the 12 week monitoring period in all groups (Sham, 1 x mTBI n = 9; 3x mTBI n = 7).

No significant differences in gross motor function were observed between the various groups over the 12 week rotarod monitoring period (Fig 3), with all groups maintaining their baseline times (120 seconds) over the complete testing period.

### Gross Neuropathology

**Fig 4:**
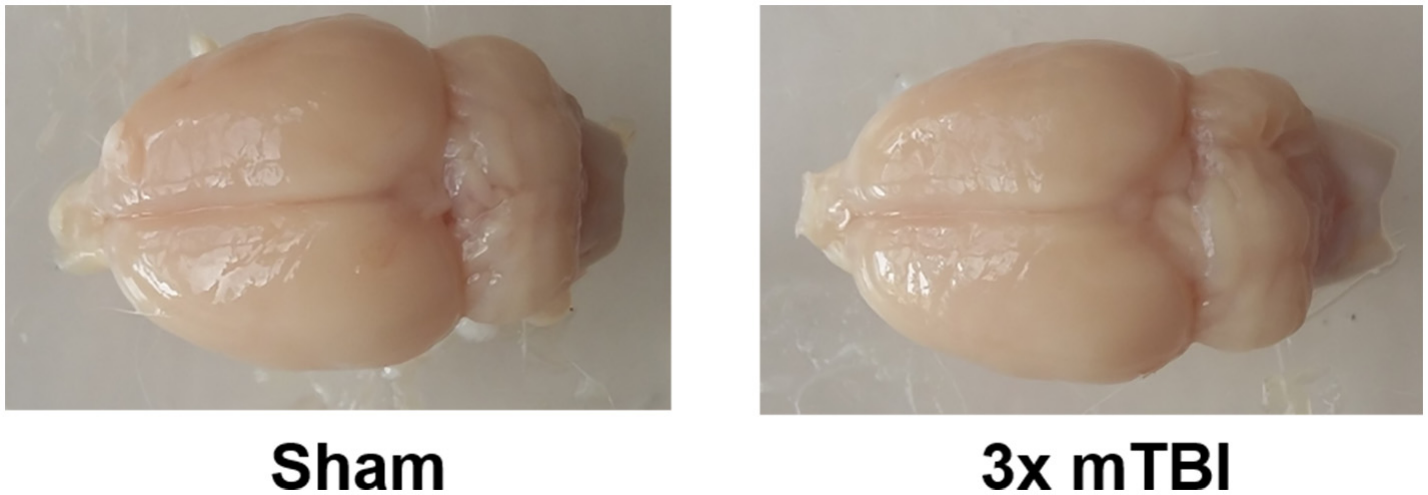
Gross Neuropathology. Gross pathology of representative rodent brains showing no lesions, hemorrhage, edema or overt tissue loss in the 3x mTBI animals compared to sham animals.

There were no gross pathological changes in the form of lesions, hemorrhage, cerebral edema or overt tissue loss observed in the 3x mTBI groups compared to shams (Fig 4).

### Immunohistochemistry

**Fig 5:**
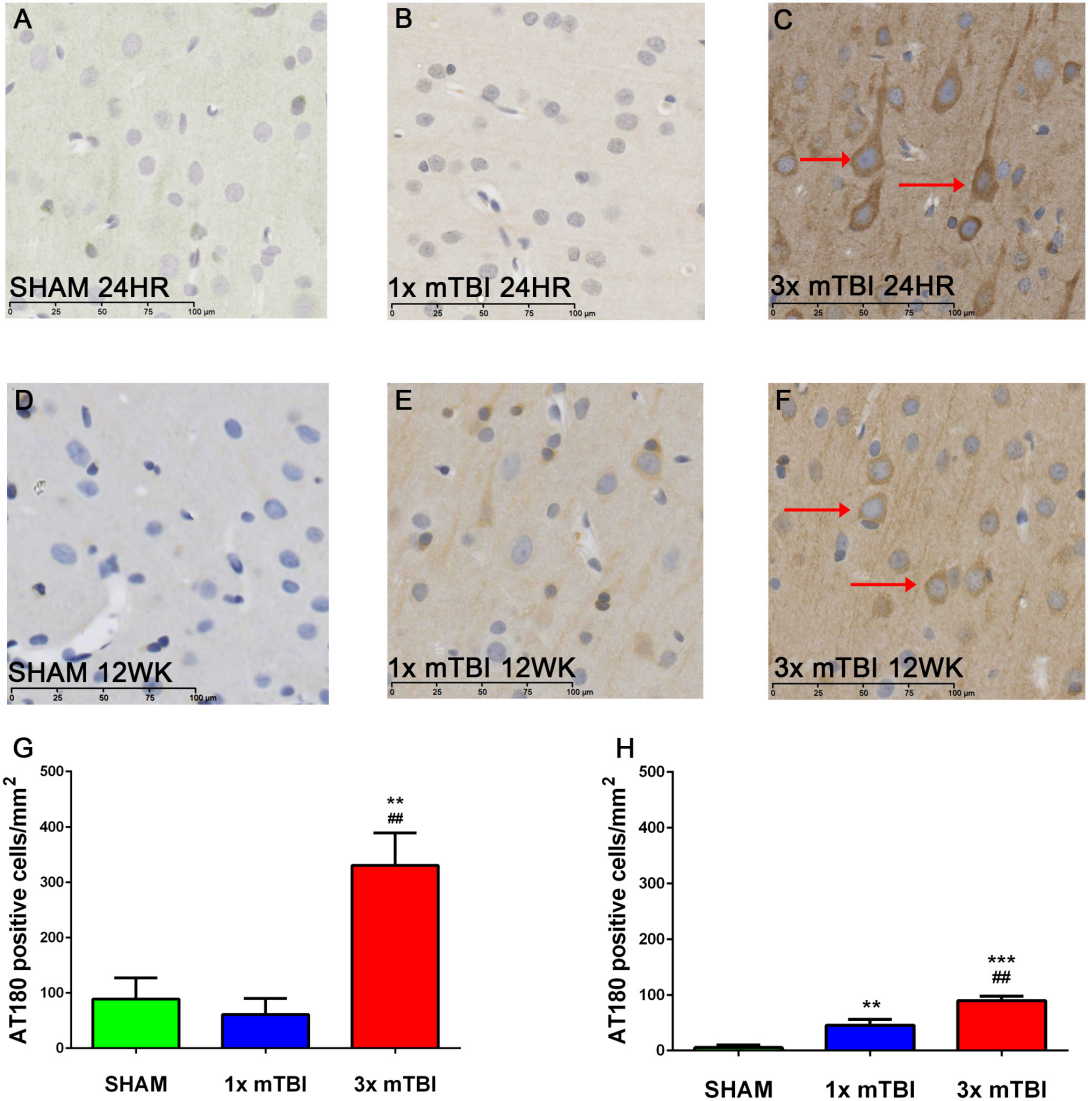
Tau Immunoreactivity post-injury. Representative images of AT180 staining within the cortex at 24 hours and 12 weeks after injury. Darker AT180 staining around cell bodies was noted at 24 hours post-injury in 3x mTBI animals (C) compared to sham (A) and 1x mTBI animals (B). Amount of AT180 immunoreactivity at 12 weeks post-injury was still increased in 3x mTBI (F) and 1x mTBI (E) compared to sham (D). This was confirmed with a count of AT180 positive cells (G-H) (***p<0.001, **p<0.01 compared to sham; ##p<0.01 compared to 1x mTBI n = 5 for 24 hour time point, n = 6 for 12 week time point)

Analysis of AT180-positive neurons within the cortex at 24 hours after injury showed a significant increase (p<0.01) in AT180 immunoreactivity within the cytoplasm of cells within the cortex in 3x mTBI (330.2±58.63) animals compared to sham (88.8±38.14) and 1x mTBI (61.25±28.77) animals (Fig 5A–5C and 5G). Although there was a decrease in overall number of AT180 positive cells, 3x mTBI animals still had a significant elevation at 12 weeks post-injury (89.58±7.81) compared to 1x mTBI (45.63±10.39; p< 0.01) and sham animals (5.33±4.40; p<0.001) (Fig 5D–5F and 5H)

**Fig 6:**
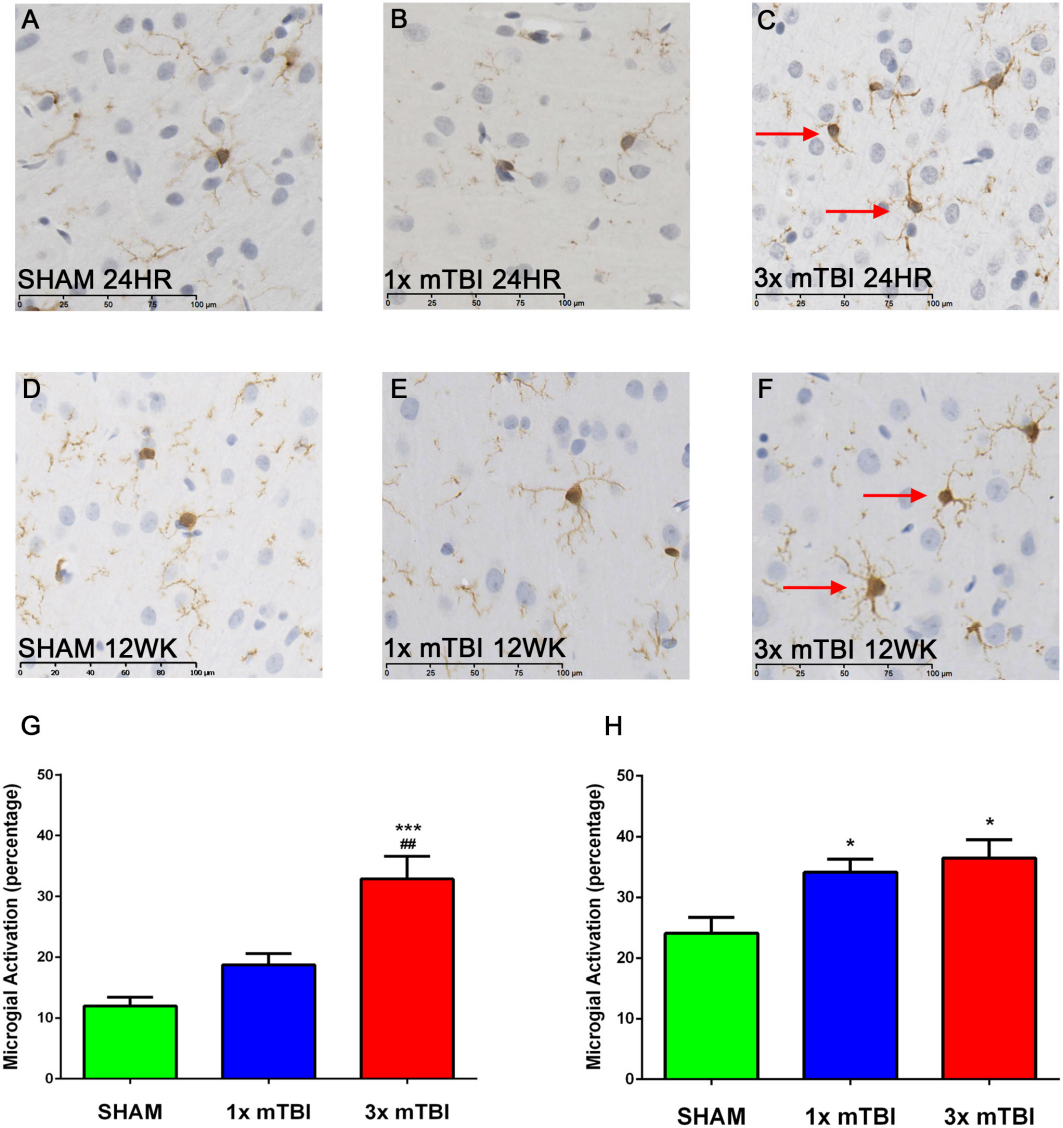
Microglial Immunoreactivity post-injury. Representative images of IBA_1_ staining in the cortex at 24 hours and 12 weeks after injury. At 24 hours post injury, increased numbers of activated microglia compared to resting were observed in 3x mTBI animals (C) compared to both sham (A) and 1x mTBI (B) groups At 12 weeks both 1x mTBI (E) and 3x mTBI (F) injury groups showing an increase in microglial activation compared to shams (D). This was confirmed via quantification determining the % of activated microglia (G). (***p<0.001, *p<0.05 compared to sham, ##p<0.01 compared to 1x mTBI, n = 5 for 24 hour time point, n = 6 for 12 week time point).

Analysis of microglial morphology in the cortex directly beneath the impact site at 24 hours post-injury (Fig 6A–6C) showed a significant increase in activated microglia in the 3x mTBI animals (32.17±5.97) compared to sham (17.71±2.88; p<0.001) and 1x mTBI animals (26.39±1.39; P<0.01) (Fig 5G). At 12 weeks post-injury (Fig 5D–5F) increased microglial activation was still evident in 3x mTBI animals (36.46±3.05) compared to shams (24.08±2.62; p<0.05) (Fig 5H). However, a significant increase (p<0.05) was also observed in the 1x mTBI group (34.12±2.15) at 12 weeks post-injury.

**Fig 7:**
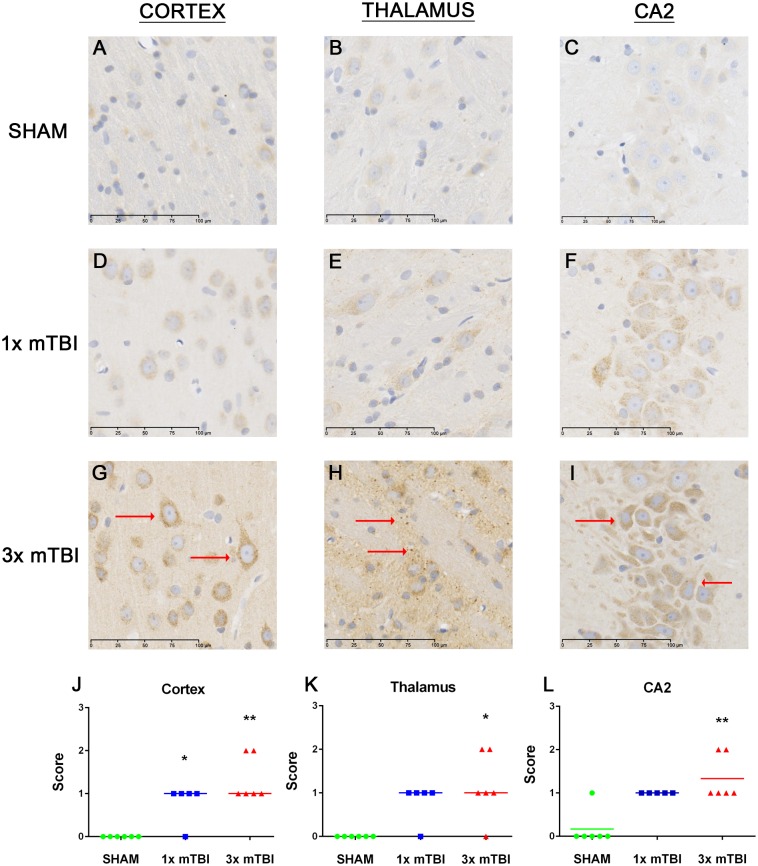
Changes in APP expression 12 weeks post-injury. Representative images of APP staining in the cortex, thalamus and CA2 region of the hippocampus in animals at 12 weeks post-injury. Increased APP immunoreactivity was observed across 3x mTBI animals (G-I, L) compared to sham (A-C, J) and 1x mTBI animals (D-F, K) (**p<0.01, *p<0.05 compared to sham, n = 6)

Significant increases (p<0.05) in APP immunoreactivity were observed at 12 weeks post- injury in 3x mTBI animals in cortical and thalamic regions, as well as the CA2 region of the hippocampus (Fig 7). 1x mTBI animals only showed a slight increase in APP immunoreactivity in the cortex, but not the thalamus or hippocampus. No changes were observed at 24 hours post-injury (S1 Fig).

## Discussion

The current study characterizes a model of repetitive mTBI that replicates key functional and histological features of clinical injury. We have demonstrated that a modified version of the Marmarou impact-acceleration model, with three mild injuries given five days apart, results in an increase in tau phosphorylation within cortical neurons in both the acute and chronic phases of injury, long-term changes in axonal pathology, as well as persistent neuroinflammation. These histological changes were associated with the development of deficits in spatial learning and memory, as seen in the Barnes Maze and the Y Maze, as well as a trend towards increased anxiety, with less time spent in the inner portion of the Open Field. Although decreased locomotor activity was noted on the Open Field, no deficits were observed in the rotarod, indicating that no gross motor deficits were present. There were no signs of overt tissue loss, contusion, edema or hemorrhage, as would be expected of a concussive injury, which typically produce no lesions that can be seen via standard imaging [26].

Appropriate and relevant experimental animal models are key to allow an understanding of the pathophysiology of the repeated injury and to allow translation of data to human patients [27]. To facilitate this a variety of animal models are required to model different aspects of the disease process, such as the types of forces used to generate a concussion. An advantage of this model is that it incorporates biomechanical criteria that have been recognized as being key when developing an experimental model of repetitive mTBI, namely that the head is struck directly with high velocity causing rapid acceleration of the head [11–13]. This generates approximately 110g of average linear force [20, 21], similar to the reported injury threshold required to produce a concussion in a human of 90g of linear acceleration force [21]. Furthermore the impact not only generates linear forces, but also rotational and angular forces [20, 21], similar to what is observed in sports injury [28]. Rotational forces are thought to be particularly important, as they generate shear strain, which is proposed to be the likely mechanisms for tissue damage following a concussion [12]. This differs from some of the pre-existing models, such as lateral fluid percussion [29] and controlled cortical impact [30], which generate a predominantly focal lesion, and involve restrictions of head movement, typically within a stereotactic frame.

A limitation of any small rodent model is that the rodent brain has structural differences compared to the human brain, as it is lissencephalic rather than gyrencephalic [31]. This is significant given that the forces associated with TBI are focused at the base of the sulci [32], where tau accumulation is most prominent in CTE [33], whereas in rodent models these forces are localized to the superficial cortical layers [32]. Furthermore there are slight differences in tau protein between rodents and human tau, with human tau having a much higher propensity to aggregate than the rodent form [34]. Despite these limitations pre-clinical models are still able to provide valuable insights into the development of neurodegeneration following repeated concussion. Of note this is one of the few models to date that have reported sub-chronic persistent increases in phosphorylated tau in non-transgenic animals following repeated concussion (reviewed in [27]), highlighting its relevance as a pre-clinical model of CTE.

Importantly this impact-acceleration model of repetitive mTBI also produces behavioral changes, including anxiety and memory deficits, similar to those seen following repeated concussion. A retrospective study of retired NFL players found that those with a self-reported history of 3 or more concussions during their playing career had a fivefold prevalence of mild cognitive impairment compared to those without a previous concussion [35]. Although CTE is currently a post-mortem diagnosis, correlation of suspected cases with their medical history and next of kin interviews, has produced a summary of symptoms suspected to be associated with the disease. Early symptoms are thought to include short-term memory loss, emotional lability and apathy, with progression to more profound cognitive deficits as the disease progresses [9, 36].

These functional changes are associated with key histopathological changes, included increases in hyperphosphorylated tau, neuroinflammation and axonal injury. Increases in phosphorylated tau both in the acute and chronic stages of injury are seen in the repetitive mTBI animals. Tau is a microtubule-associated protein that is involved with providing structural support to neuronal cells within the CNS [37]. The ability of tau to bind to the microtubules is maintained by its level of phosphorylation. If tau becomes hyperphosphorylated, the ability to stimulate microtubule assembly is inhibited [38], leading to detachment of the hyperphosphorylated tau from the microtubules which makes it prone to self-aggregation and polymerization [39]. This leads to the formation of tau oligomers which can further aggregate to form paired helical filaments (PHFs) which then assemble to form neurofibrillary tangles (NFTs) as seen in AD and CTE [40, 41]. TBI, particularly repeated concussion, has been shown to influence tau hyperphosphorylation both pre-clinically and clinically [42–45]. Human serum analysis following mTBI has also shown increased levels of phosphorylated tau 6 hours post injury, suggesting that these processes can occur early within the disease process [46]. Of note this alteration in tau dynamics may contribute to secondary injury following TBI. A study by Gerson et al. (2016) demonstrated that TBI-derived tau oligomers worsened cognition and accelerated pathology development when injected into the hippocampi of mice overexpressing human tau [47]. Furthermore complete ablation or partial reduction of tau prevented deficits in spatial memory and learning on the Barnes Maze, with a decreased latency to the target following repeated mild frontal impact [48].

Another key feature seen following repeated concussion is a persistent neuroinflammatory response. Specifically, the present study found that repetitive mTBI increased the expression of activated microglia in both the acute and chronic phases of injury. Although it is widely thought that the inflammatory changes observed in mTBI are completely reversible with symptoms typically resolving with time, the synergistic effect of repetitive mild injuries over a short amount of time exacerbates this inflammatory response [5, 49, 50]. As such, there is the potential for an exponential increase in the inflammatory cascade following mTBI [5] and for the neuroinflammatory response to persist chronically. Increases in chronic neuroinflammation have been observed in the brains of young athletes exposed to repetitive mTBI and displaying CTE-like pathologies post-mortem [51]. Neuroinflammation following TBI has also been implicated in the pathogenesis of other neurodegenerative disorders including Alzheimer’s disease [52], with this neuroinflammation not resolving for years following the initial insult [53]. Other existing animal models of repetitive mTBI have also reported increases in neuroinflammation following injury, with increased GFAP reactivity and serum levels of the pro-inflammatory cytokine interleukin-6 (IL-6) observed in a mouse weight drop model [54], while increases in microglial activation have been reported in a fluid percussion injury model of repetitive mTBI [19].

The changes in axonal integrity observed long-term are also worth noting given that axonal injury is present in many other models of repetitive mTBI [27, 55] and in human clinical cases [56, 57]. Axonal injury is thought to play a key role in promoting abnormal tau phosphorylation, and the high strain placed on axons in TBI means that tau proteins behave more rigidly than usual. This may inhibit the ability of adjacent microtubules to slide past each other in response to axonal stretching, resulting in the subsequent rupture [58]. As tau becomes unbound from the microtubule it facilitates phosphorylation at disease related sites [59–62], and promotes aggregation into oligomers and NFTs [63]. Although changes in axonal integrity were observed within the cortical, thalamic and hippocampal regions in this model, further investigation into specific regional differences, particularly within the hippocampus, is warranted. Further investigation into acute and chronic changes in synaptic transmission and other markers of axonal integrity will also further elucidate the extent of these disease processes, particularly within the acute phase where in the present study, no changes were observed.

It is important to note that there were transient changes observed in the single injury mTBI group, both behaviorally and pathologically. This is consistent with data from clinical studies suggesting that patients with a single mTBI show increased depressive and anxiety-like symptoms as well as global brain atrophy, particularly in the white matter regions of the cingulum and cingulate gyrus, up to one year post-injury [64]. The most common symptoms observed clinically following a single mTBI include cognitive deficits in the acute stage, typically resolving within months of the initial injury [65]. Given the prevalence of post-concussive symptoms such as headache persisting for weeks to months following a single injury, the data presented within this study and others suggest that a single mTBI is enough to cause long term pathological changes within the brain [66]. Animal models have demonstrated that a single mTBI can acutely induce neurodegenerative and neuroinflammatory changes that increase with injury severity, and that this is intensified with repeated trauma [67–69]. This suggests that although a single injury is enough to cause some acute and chronic changes, repetitive injuries appear to exacerbate these processes.

In conclusion, this study characterizes a clinically relevant, *in vivo* model of repetitive mTBI that produces the key short- and long-term pathological and functional changes observed in clinical concussive injury, and the typical hallmarks of progressive CTE. Accordingly, this model of repetitive impact-acceleration mTBI represents a useful experimental approach to explore the underlying pathogenesis of neurodegeneration following repeated concussion, and to develop potential therapies.

## Supporting Information

S1 Fig APP Immunoreactivity 24 hours post-injury. Representative images of APP staining in the cortex, thalamus and CA2 region of the hippocampus in animals at 24 hours post-injury. No changes were observed between sham, 1x mTBI and 3x mTBI groups (n = 5 per group).
(TIF)
