# Supplementary figures and images for: Short and Long Term Behavioral and Pathological Changes in a Novel Rodent Model of Repetitive Mild Traumatic Brain Injury
Source: PLoS One. 2016 Aug 9;11(8):e0160220. doi: 10.1371/journal.pone.0160220 (PMC4978416; doi:10.1371/journal.pone.0160220)

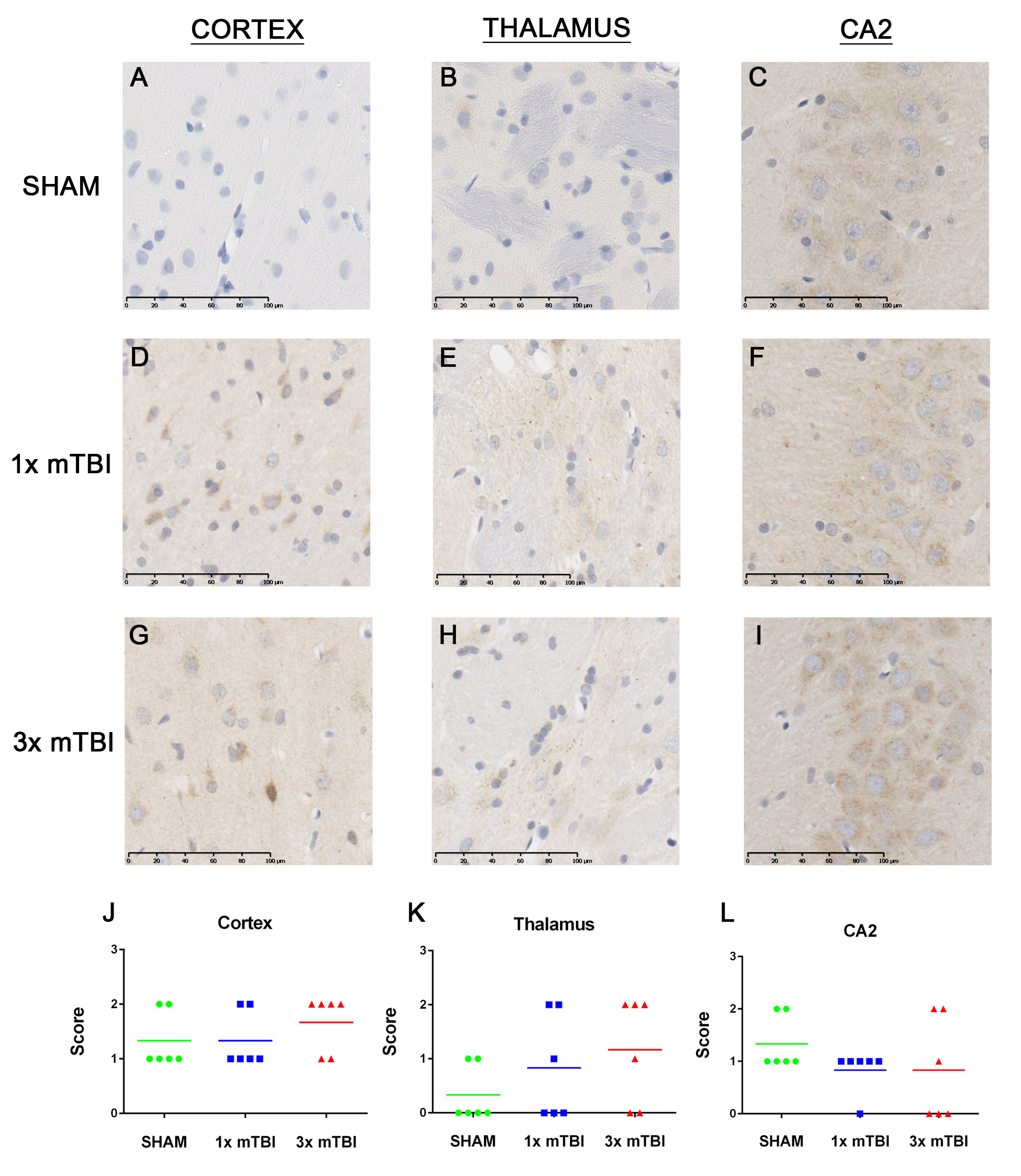

Supplement: S1 Fig — Representative images of APP staining in the cortex, thalamus and CA2 region of the hippocampus in animals at 24 hours post-injury. No changes were observed between sham, 1x mTBI and 3x mTBI groups (n = 5 per group). (TIF) [file pone.0160220.s001.tif]
